# Control of cytokine-driven eosinophil migratory behavior by TGF-beta-induced protein (TGFBI) and periostin

**DOI:** 10.1371/journal.pone.0201320

**Published:** 2018-07-26

**Authors:** Karina T. Barretto, Calvin M. Swanson, Christopher L. Nguyen, Douglas S. Annis, Stephane J. Esnault, Deane F. Mosher, Mats W. Johansson

**Affiliations:** 1 Department of Biomolecular Chemistry, University of Wisconsin, Madison, Wisconsin, United States of America; 2 Department of Medicine, University of Wisconsin, Madison, Wisconsin, United States of America; Universidade Federal de Juiz de Fora, BRAZIL

## Abstract

Periostin, which is induced by interleukin (IL)-13, is an extracellular matrix (ECM) protein that supports α_M_β_2_ integrin-mediated adhesion and migration of IL-5-stimulated eosinophils. Transforming growth factor (TGF)-β-induced protein (TGFBI) is a widely expressed periostin paralog known to support monocyte adhesion. Our objective was to compare eosinophil adhesion and migration on TGFBI and periostin in the presence of IL-5-family cytokines. Eosinophil adhesion after 1 h and random motility over 20 h in the presence of various concentrations of IL-5, IL-3, or granulocyte macrophage-colony stimulating factor (GM-CSF) were quantified in wells coated with various concentrations of TGFBI or periostin. Results were compared to video microscopy of eosinophils. Cytokine-stimulated eosinophils adhered equivalently well to TGFBI or periostin in a coating concentration-dependent manner. Adhesion was blocked by anti-α_M_β_2_ and stimulated at the lowest concentration by GM-CSF. In the motility assay, periostin was more potent than TGFBI, the coating-concentration effect was bimodal, and IL-3 was the most potent cytokine. Video microscopy revealed that under the optimal coating condition of 5 μg/ml periostin, most eosinophils migrated persistently and were polarized and acorn-shaped with a ruffling forward edge and granules gathered together, in front of the nucleus. On 10 μg/ml periostin or TGFBI, more eosinophils adopted a flattened pancake morphology with dispersed granules and nuclear lobes, and slower migration. Conversion between acorn and pancake morphologies were observed. We conclude that TGFBI or periostin supports two modes of migration by IL-5 family cytokine-activated eosinophils. The rapid mode is favored by intermediate protein coatings and the slower by higher coating concentrations. We speculate that eosinophils move by haptotaxis up a gradient of adhesive ECM protein and then slow down to surveil the tissue.

## Introduction

The extracellular matrix (ECM) protein periostin [1] is upregulated by type 2 immunity mediators such as IL-13 in the asthmatic airway [2]. Substrate-bound purified periostin is known to support adhesion of IL-5- IL-3-, or GM-CSF-stimulated eosinophils and migration of IL-5-stimulated eosinophils [3–6]. Adhesion of IL-5-activated eosinophils to periostin is mediated by α_M_β_2_ integrin [3, 5, 6]. We have suggested, therefore, that periostin is a mediator of eosinophil enrichment and persistence in the asthmatic airway [1, 3, 4, 7].

Transforming growth factor (TGF) β-induced protein (TGFBI, βig-h3) is a paralog of periostin and, like periostin, contains an amino (N)-terminal cysteine-rich sequence and four fasciclin-1 (FAS1) modules [1]. The disulfide pattern of TGFBI was recently determined [8]. Inter-module bridges between the cysteine-rich sequence and the second FAS1 module and between the first two FAS1 modules indicate that the N-terminal cysteine-rich sequence and the first two FAS1 modules adopt a complicated fold, possibly leaving the third and fourth FAS1 modules exposed [8]. This model should be applicable to periostin, which contains the same set of cysteine residues [1]. Unlike periostin, TGFBI lacks an alternatively spliced tail carboxy (C) terminal to the fourth FAS1 module [1]. TGFBI is an ECM protein that is widely expressed, including in the lung, and is induced by TGF-β in bronchial myofibroblasts and other fibroblasts [1, 9, 10]. Genetic deletion of TGFBI is not lethal but has multiple effects, including defective development of alveolar structure and function in mice [11]. Although studies comparing and contrasting activities of TGFBI and periostin head-to-head are sparse, TGFBI and periostin were recently found to act similarly in the heart in affecting fibrosis and disease responsiveness; however, TGFBI is seemingly not necessary in the heart after myocardial infarction injury and is fully compensated by the more prominently expressed effector periostin [12].

TGFBI is known to support α_M_β_2_-integrin-mediated adhesion of monocytes [13]. We asked whether TGFBI adsorbed in a microtiter plate, like adsorbed periostin, supports eosinophil adhesion and migration induced by IL-5 family cytokines in short-term adhesion and long-term bead-clearing migration assays, which model adhesion to and migration on ECM molecules. To complement the bead-clearing assay, we examined the migratory behavior of eosinophils on TGFBI or periostin adsorbed on glass by video differential interference contrast (DIC) microscopy. We report that TGFBI and periostin are both adhesive ligands for eosinophil α_M_β_2_ integrin that may be important for eosinophil recruitment and retention in ECM, and that IL-5 family cytokines acting on eosinophils have different potencies for adhesion and migration and potentially play different roles at various times after eosinophil activation. We observed two morphologies and modes of migration of activated eosinophils on TGFBI or periostin that are pertinent to the migration results: rapidly-migrating polarized, acorn-shaped cells, which tended to be most frequent on intermediate periostin coating, and slower flatter, pancake-shaped cells most frequent on high protein coating.

## Materials and methods

### Eosinophils

Eosinophils were obtained and purified from heparinized blood of donors with allergy and with or without mild allergic asthma by negative selection using a cocktail of anti-CD16, anti-CD14, anti-CD3, and anti-glycophorin beads as before [4]. Subjects with prescriptions for low doses of inhaled corticosteroids (ICS) did not use their ICS on the day of the blood draw. The purity and viability of eosinophils were ≥ 98% [4]. The studies were approved by the University of Wisconsin-Madison Health Sciences Institutional Review Board (protocol No. 2013–1570). Informed written consent was obtained from each subject before participation.

### TGFBI and periostin

Human TGFBI (Uniprot identifier No. Q15582) complementary DNA (cDNA) was obtained by reverse transcription of RNA isolated from MG-63 cells using M-MLV reverse transcriptase (Promega, Madison, WI, USA) and a TGFBI-specific oligonucleotide (5’ tgc att cct cct gta gtg c 3’) coding for a sequence at the beginning of the 3’ untranslated region. The cDNA was used as template for polymerase chain reaction (PCR) and cloned into pAcGP67.coco [14] using standard molecular biology techniques. The complete insert sequence was verified before expression. TGFBI was produced as a secreted protein by insect cells using a baculovirus system and purified as described [14]. PN-S, the shortest splice variant of human periostin containing the N-terminal cysteine-rich sequence and the four FAS1 modules but lacking variably spliced C-terminal sequences encoded by exons 17, 18, 19, and 21 (Uniprot No. Q15063-7), was produced in insect cells as before [3, 4, 15]. Recombinant human TGFBI and longest periostin variant (PN-L) (Uniprot No. Q15063-1) [3, 4], expressed by mouse NSO cells, were purchased from R&D Systems (Minneapolis, MN, USA).

### Antibodies and other reagents

Inhibitory monoclonal antibodies (mAbs) to integrin subunits (anti-α_M_ clone 2LPM19c and anti-β_2_ TS1/18) were from Santa Cruz Biotechnology (Dallas, TX, USA) and BioLegend (San Diego, CA, USA), respectively. Isotype control mouse IgG_1_ (clone MOPC-21) was from BD Biosciences (Franklin Lakes, NJ, USA). Recombinant human IL-5, IL-3, and GM-CSF were from R&D Systems. 4’,6-diamidino-2-phenylindole (DAPI) was from Life Technologies (Eugene, OR, USA).

### Cell adhesion assay

Eosinophil adhesion for 1 h was quantified using an eosinophil peroxidase assay as described [3], with the following modifications. After eosinophils were resuspended at 2 x 10^6^/ml in Roswell Park Memorial Institute (RPMI) medium with 0.2% bovine serum albumin (BSA), cells were equilibrated for 1 h at 37°C [16] before dilution and addition to coated wells. Further, the colored eosinophil peroxidase product was measured in a SpectraMax M5 plate reader (Molecular Devices, Sunnyvale, CA, USA).

### Cell motility assay

Motility of eosinophils, equilibrated as in the cell adhesion assay, was assessed in a bead clearing assay that ran for 20 h with wells viewed in an Eclipse Ti inverted microscope (Nikon, Melville, NY, USA) and images acquired and quantified as before [4].

### Differential interference contrast (DIC) video microscopy

Glass bottom culture 35 mm diameter Petri dishes with 14 mm glass diameter No. 1.0 coverslip (MatTek, Ashland, MA, USA) were used. The glass area was coated with 350 μl protein (5 or 10 μg/ml) in Tris-buffered saline (TBS), pH 7.4, for 2 h at 37°C, washed with TBS, blocked with neat fetal bovine serum (FBS) for 30 minutes at 37°C, and washed with TBS. Eosinophils, resuspended at 2 x 10^6^/ml in RPMI with 10% FBS and equilibrated as above, were diluted in the same medium to 2 x 10^5^/ml, and 2 ml of this cell suspension was added to the dish. Two μl of IL-5 stock solution (50 μg/ml) was added, yielding a final concentration of 50 ng/ml. Cells were viewed in a DMi8 inverted wide-field microscope (Leica, Buffalo Grove, IL, USA) with a motorized stage and Tokei Hit temperature- and CO_2_-controlled chamber using a 63x oil immersion objective, at the University of Wisconsin-Madison Optical Imaging Core facility. Five minute videos with 1 frame/s were acquired up to 35 minutes after addition of IL-5, and videos and individual images were exported, using Leica Application Suite X (LASX) software. Cells defined as having an acorn-shaped morphology were polarized with a ruffling leading edge and pseudopods, the nuclear lobes at the rear, and the granules closely gathered together between the forward edge and the nucleus and moving as a unit in a coordinated manner. Cells defined as having a “pancake-shaped” morphology were flatter, more spread with dispersed granules and nuclear lobes. Cells defined as unactivated were round, not polarized, not flat, and not spread. Cell area was quantified using Fiji software (http://fiji.sc/Fiji).

### Statistical analysis

Student’s *t* test was used to compare data between two groups. This was complemented for some experiments by analysis of variance (ANOVA) with Dunnett’s or Tukey’s multiple comparisons post test in order to compare data among groups. *P* ≤ 0.05 was considered significant. Analyses were performed using Prism (GraphPad, San Diego, CA, USA).

## Results

### TGFBI supports eosinophil adhesion with similar coating concentration dependence as periostin variants

In order to determine whether TGFBI supports eosinophil adhesion and compare it to periostin, assays were performed in microtiter plates in which wells were coated with different concentrations of recombinant human TGFBI commercially available from R&D Systems or produced in insect cells in our laboratory, the longest human periostin splice variant purchased from R&D (PN-L), or the shortest human periostin splice variant (PN-S) produced in insect cells. Wells were post-coated with neat fetal bovine serum (FBS). Control wells were coated only with FBS.

**Fig 1:**
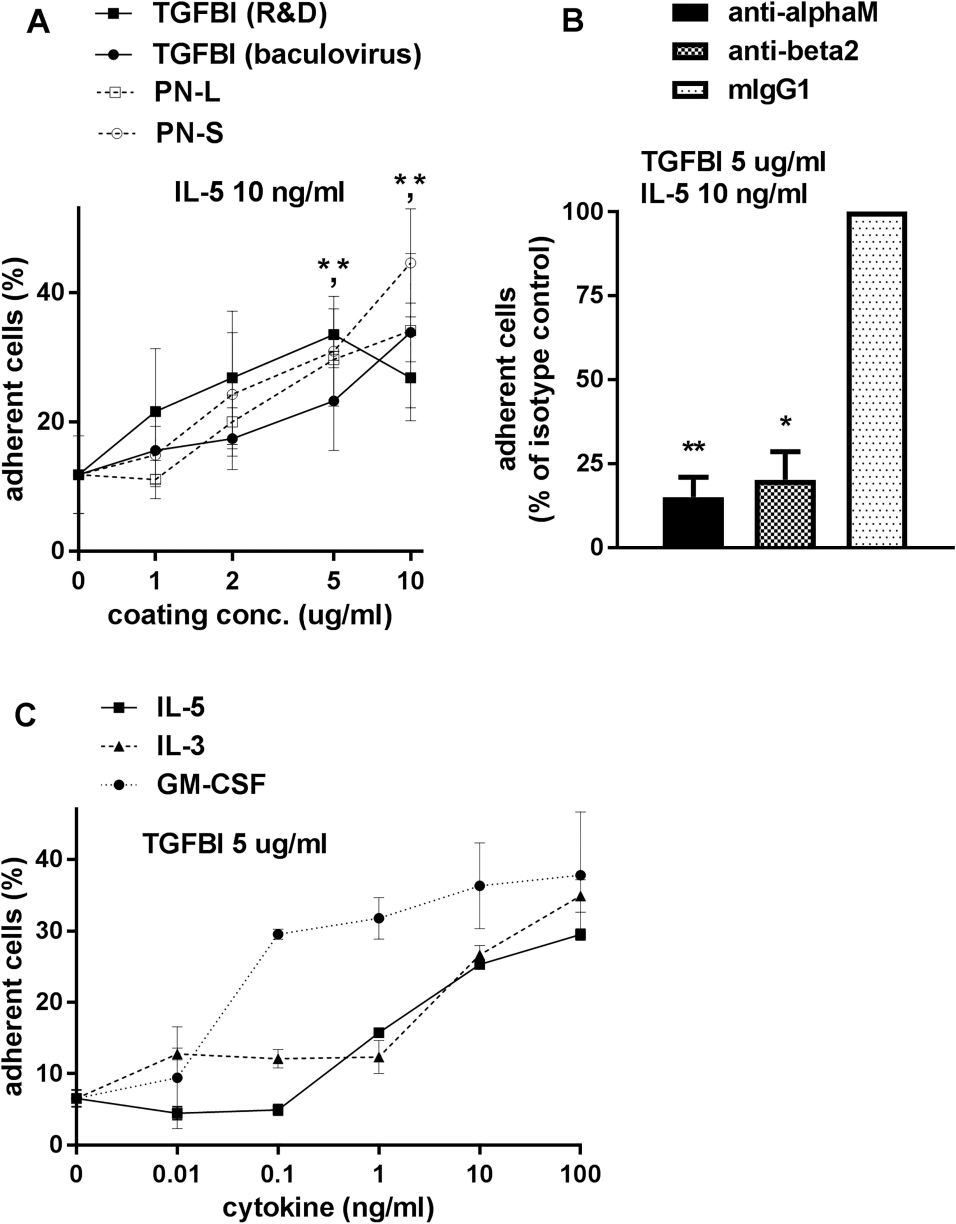
Eosinophil adhesion to TGFBI and periostin; effects on adhesion to TGFBI of mAbs and cytokines. Adhesion of IL-5-activated eosinophils (A) to TGFBI and periostin, (B) effects on eosinophil adhesion to TGFBI by anti-α_M_ or anti-β_2_ integrin monoclonal antibody (mAb), and (C) eosinophil adhesion to TGFBI in the presence of different cytokines. Adhesion of purified blood eosinophils incubated for 1 h in the presence of IL-5, 10 ng/ml (A and B), or in the presence of different concentrations of IL-5 IL-3, or GM-CSF (C) in wells of microtiter plates. (A) Wells were coated with the following proteins at different concentrations: transforming growth factor (TGF)-β-induced protein (TGFBI) purchased from R&D Systems, TGFBI produced using baculovirus, the longest periostin splice variant (PN-L) from R&D Systems, and the shortest periostin splice variant lacking alternatively spliced C-terminal sequences (PN-S) produced using baculovirus. Wells were post-coated with FBS. Symbols and brackets: mean ± standard error of the mean (SEM), n = 3 donors; *P < 0.05 versus FBS post-coat alone (for R&D TGFBI or PN-L at 5 μg/ml and for baculovirus TGFBI or PN-S at 10 μg/ml). ANOVA with Dunnett’s post test confirmed *P* < 0.05 for R&D TGFBI and PN-S at 5 and 10 μg/ml, respectively. Further, ANOVA showed no significant difference among the four proteins at each coating concentration. (B) Wells were coated with R&D TGFBI at 5 μg/ml followed by post-coat with FBS. Eosinophils were preincubated with mAb (10 μg/ml) to the indicated integrin subunit. Mean ± SEM, n = 2 donors, **P < 0.01, *P < 0.05 versus isotype control. ANOVA confirmed significant difference (*P* < 0.01) among treatments and Dunnett’s post test confirmed significant difference (*P* < 0.01) of each of the mAbs versus isotype control. The number of adherent cells with isotype control was 27% ± 15%. (C) Wells were coated with R&D TGFBI at 5 μg/ml followed by post-coat with FBS. Mean ± SEM of two wells per treatment in one representative experiment (of two). In addition, similar dose response curves were obtained in two experiments in which wells were coated with 2 μg/ml TGFBI.

R&D and insect cell TGFBI supported adhesion at 1 h of eosinophils stimulated with IL-5 (10 ng/ml) compared to the FBS control in a manner dependent on the coating concentration (Fig 1A). R&D PN-L or insect cell PN-S also supported adhesion of IL-5-stimulated eosinophils in a coating concentration-dependent manner (Fig 1A), as we found previously [3]. The coating concentration dependences of R&D TGFBI, baculovirus TGFBI, PN-L, and PN-S were similar, with no significant difference among the four proteins at each coating concentration (Fig 1A).

### Eosinophil adhesion to TGFBI is mediated by α_M_β_2_ integrin

Pre-incubation of IL-5-treated eosinophils with inhibitory mAbs 2LPM19c and TS1/18 to α_M_ and β_2_ integrin subunits, respectively, inhibited eosinophil adhesion to TGFBI (Fig 1B). This inhibition of adhesion by anti-α_M_ or anti-β _2_ was similar to that on periostin [3].

### GM-CSF stimulates eosinophil adhesion to TGFBI with a different dose-response curve than does IL-5 or IL-3

The stimulating effect of IL-5 on eosinophil adhesion to TGFBI at 1 h was dose dependent (Fig 1C). The IL-5 family cytokines IL-3 and GM-CSF also stimulated adhesion to TGFBI (Fig 1C). IL-3 stimulated adhesion with a similar dose-response curve as did IL-5 (Fig 1C). The dose-response curve of GM-CSF was shifted left compared to those of IL-5 or IL-3, with GM-CSF being clearly active down to 0.1 ng/ml; thus, GM-CSF was active at a lower concentration than IL-5 or IL-3 (Fig 1C). These curves were similar to those for cytokine-stimulated eosinophil adhesion to periostin, on which GM-CSF was also active down to 0.1 ng/ml [3].

### TGFBI supports motility of eosinophils but is less effective than periostin

**Fig 2:**
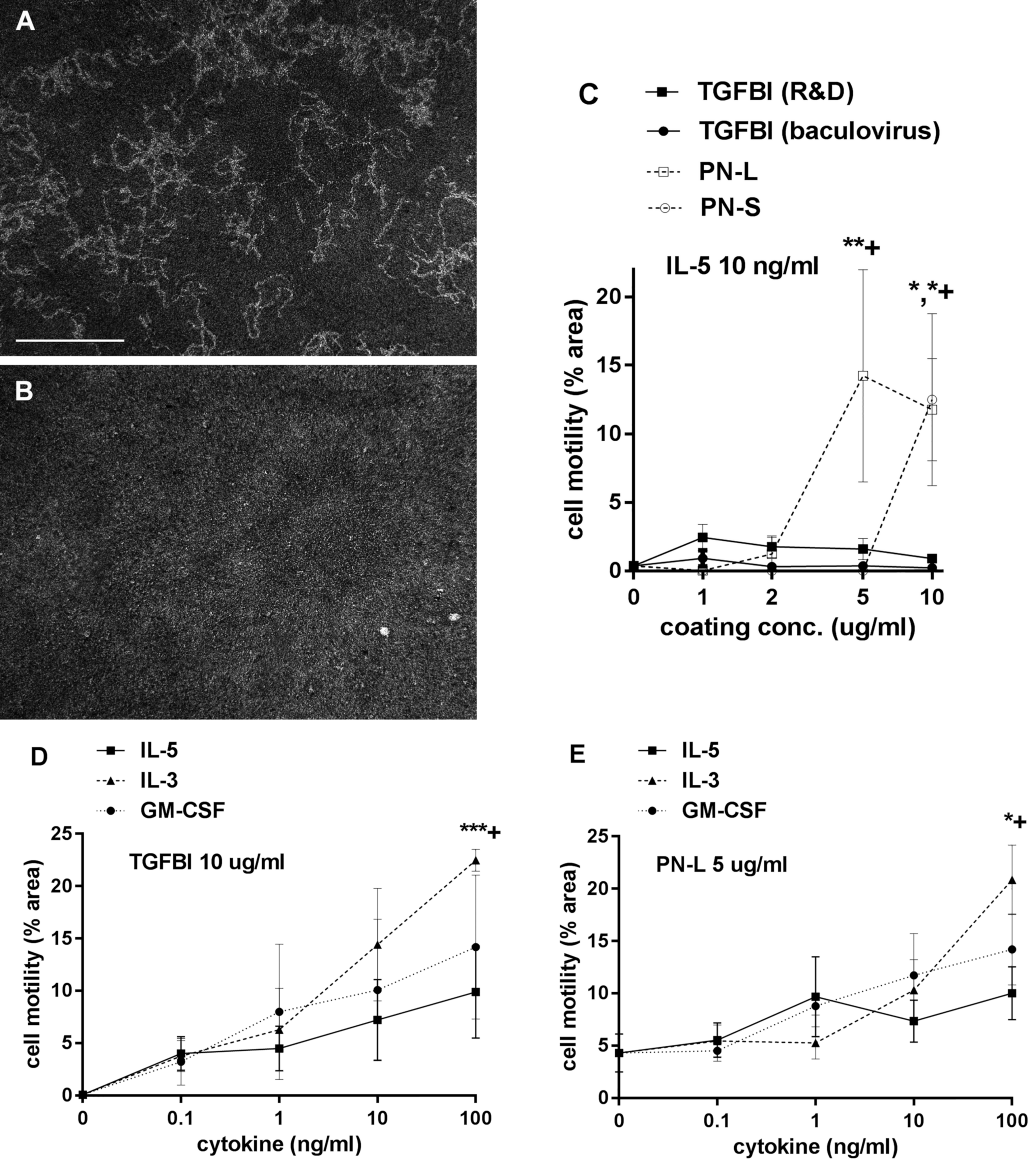
Eosinophil motility on TGFBI and periostin; effects of coating and cytokine concentrations. Microbead monolayer assay for cell motility (A, B), and eosinophil motility on TGFBI and periostin variants in the presence of IL-5, 10 ng/ml (C) and on TGFBI (D) and periostin (E) in the presence of different cytokines. (A, B) Paths of migrating eosinophils (in the presence of IL-5, 10 ng/ml) were revealed by perturbation of a monolayer of 1 μm-diameter latex beads. Wells were photographed after 20 h. Morphometric analysis of track areas was performed using Fiji. Positive (A) or negative (B) examples are shown. (A) PN-L coated at 5 μg/ml followed by post-coat with FBS, motility 16.1% of area. (B) FBS post-coat only, motility 0.1% of area. Bar, 500 μm. (C) Motility of eosinophils in wells coated with different concentrations of R&D TGFBI, baculovirus TGFBI, PN-L, or PN-S in the presence of IL-5, 10 ng/ml. Symbols and brackets: mean ± SEM, n = 3 donors, **P < 0.01 for PN-L at 5 μg/ml, *P < 0.05 for PN-L or PN-S at 10 μg/ml versus FBS post-coat alone; +*P* < 0.05 for PN-L versus PN-S at 5 μg/ml or for PN-L or PN-S versus TGFBI at 10 μg/ml. (D, E) Motility of eosinophils in wells coated with R&D TGFBI, coated at 10 μg/ml (D), or PN-L, coated at 5 μg/ml (E), in the absence or presence of different concentrations of IL-5, IL-3, or GM-CSF. Symbols and brackets: mean ± SEM, n = 3 donors for TGFBI (D), 4 donors for PN-L (E); ***P < 0.001, *P < 0.05 for IL-3 versus no cytokine; +*P* < 0.05 for IL-3 versus IL-5. ANOVA resulted in *P* = 0.01 for difference among IL-3 concentrations in (D) and Dunnett’s post test confirmed significant difference (*P* < 0.01) for IL-3 100 ng/ml versus no cytokine and gave *P* < 0.05 for IL-3 10 ng/ml versus no cytokine in (D). ANOVA resulted in *P* < 0.01 for difference among IL-3 concentrations in (E) and Dunnett’s post test confirmed signficiant difference (*P* < 0.01) for IL-3 100 ng/ml versus no cytokine in (E).

The effect of TGFBI on surface-dependent motility (haptokinesis) of eosinophils over 20 h was assessed with a microbead monolayer assay [3, 4]. After protein coating and blocking, microbeads were added at a concentration that, after centrifugation of the plate, forms a nearly confluent “lawn” of beads. In Fig 2A, a positive example with PN-L coated at 5 μg/ml, white areas cleared of microbeads represent paths of migration in contrast to the dark bead monolayer background. Fig 2B shows a negative example with a control well only coated with neat FBS. Motility was quantified as cleared area of tracks using Fiji software [4]. Immobilized TGFBI supported random motility of IL-5-stimulated eosinophils (Fig 2D). However, the motility on TGFBI in the presence of 10 ng/ml IL-5 was variable among donors (compare the three donors in Fig 2D with a mean of 7% of area cleared and the three donors in Fig 2C with 1% under the same experimental conditions). In a comparison using the same donors, TGFBI was less effective in supporting motility than PN-L or PN-S in the presence of 10 ng/ml IL-5 (Fig 2C). At a 10 μg/ml coating concentration, PN-S or PN-L were more effective in supporting eosinophil motility than TGFBI (Fig 2C). Further, at 5 μg/ml, PN-L was more effective than PN-S (Fig 2C). Motility on PN-L was maximal at this concentration (5 μg/ml) (Fig 2C), as we have found before [3].

### IL-3 stimulates eosinophil motility on TGFBI or periostin to a higher degree than does IL-5 or GM-CSF

The stimulating effect of IL-5 on eosinophil motility to TGFBI (Fig 2D) or PN-L (Fig 2E) over 20 h was dose-dependent. IL-3 and GM-CSF also stimulated motility on TGFBI (Fig 2D) and PN-L (Fig 2E). IL-3 stimulated motility up to a higher maximal level than did IL-5 or GM-CSF on TGFBI or PN-L, with IL-3 having a greater effect than IL-5 on both proteins (Fig 2D and 2E). In addition, we observed that in the absence of cytokine (i.e., at 0 ng/ml), there was no or essentially no motility on TGFBI (Fig 2C and 2D), whereas there was, as we have noted before [3], a low degree of motility on PN-L (Fig 2E).

### Video microscopy of eosinophil motility on TGFBI or periostin

**Fig 3:**
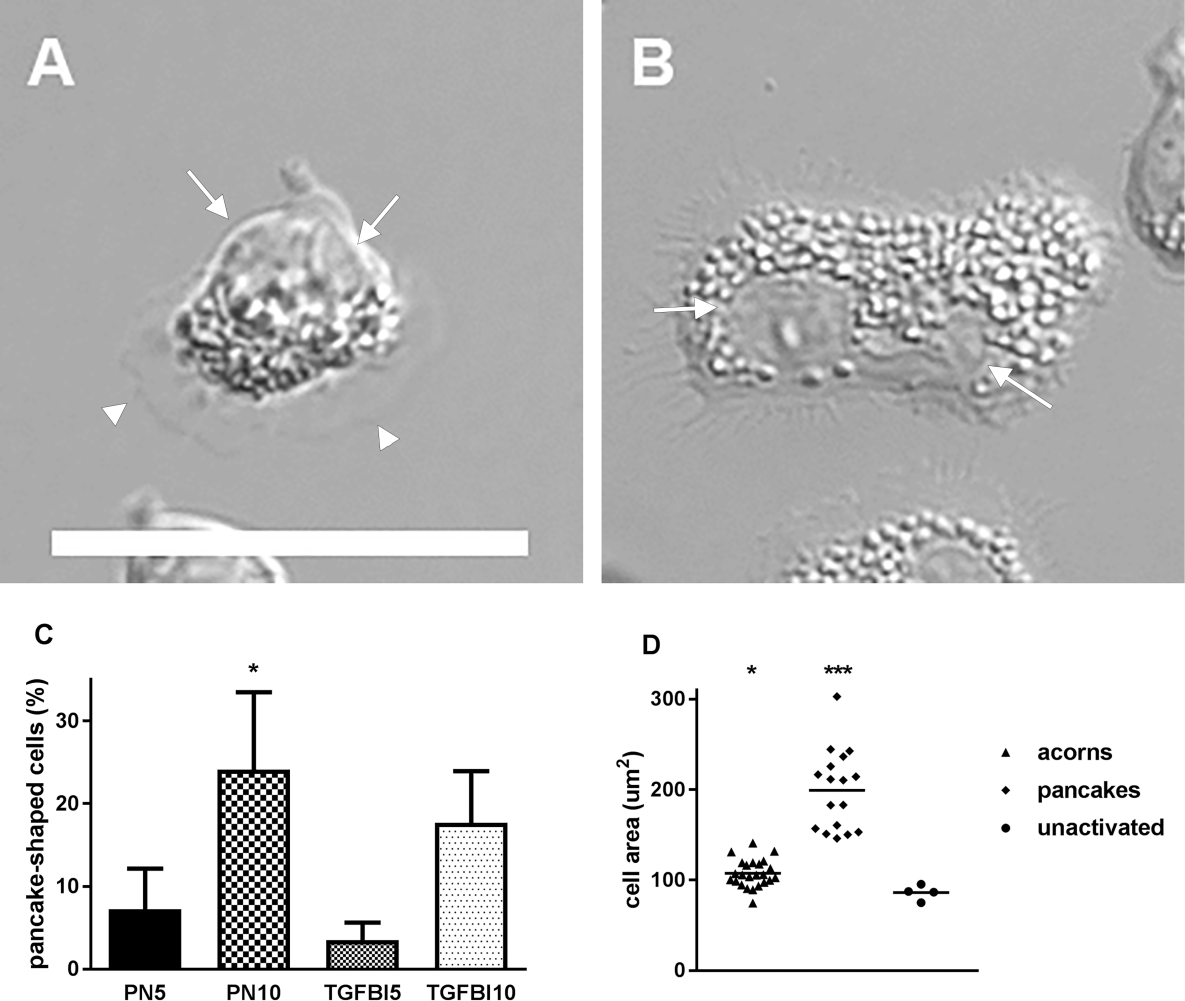
Morphology of eosinophils migrating on TGFBI or periostin. Morphology of eosinophils migrating on TGFBI or periostin in the presence of IL-5, as observed by DIC video microscopy. Examples of eosinophils with (A) acorn-shaped or (B) pancake-shaped morphology, Bar, 25 μm. Arrows, nuclear lobes; arrowheads, periphery of ruffling edge. (C) Percentage of eosinophils with pancake-shaped morphology on PN-L or R&D TGFBI, coated at 5 or 10 μg/ml, in the presence of IL-5, 50 ng/ml, after 5–10 minutes. Symbols and brackets: mean ± SEM, n = 5 donors with a video with 6–19 cells scored for each condition; *P < 0.05 (versus PN-L at 5 μg/ml). (D) Quantification of area of cells with the different morphologies. A frame from the middle time point of each of the four movies in Supporting Information S1–S4 Movies were used. Cell area was quantified using Fiji. Bars, means; ***P < 0.001 versus acorn-shaped or unactivated, *P < 0.05 versus unactivated. ANOVA confirmed significant difference (*P* < 0.001) among morphologies and Tukey’s post test confirmed significant difference (*P* < 0.001) of pancake-shaped versus acorn-shaped or unactivated.

Video microscopy with differential interference contrast optics was performed up to 35 minutes after addition of IL-5, to compare further the effects of adsorbed TGFBI and periostin, and of two different coating concentrations (5 and 10 μg/ml), on eosinophil motility, and to examine the morphology of migrating eosinophils and mode of migration. The majority of eosinophils migrating on TGFBI or periostin had a polarized morphology and were acorn-shaped (Fig 3A and S1–S4 Movies, particularly S1 Movie). The morphology was somewhat similar to eosinophils that have been activated by IL-5 in suspension and examined after cytospinning [16], except that the migrating cells had a ruffling leading edge with pseudopods (Fig 3A and S1–S4 Movies). This morphology tended to be most frequent on periostin coated at 5 μg/ml (82% ± 7% of the cells, mean ± standard error of the mean [SEM], n = 5, at 5–10 minutes) (see S1 Movie). Migration of acorn-shaped IL-5-activated eosinophils was persistent and occurred with the granules closely clustered together and moving as a unit in a coordinated manner, and the nucleus and trailing tail at the rear (S1–S4 Movies, particularly S1 Movie). Acorn-shaped eosinophils had a mean area of 108 μm^2^ (Fig 3D). The migration velocity of acorn-shaped eosinophils on periostin coated at 5 μg/ml was 0.2 μm/s. Some cells had a flatter, more spread, and “pancake-shaped” morphology with dispersed granules and nuclear lobes (Fig 3B, S1–S4 Movies, particularly S2 and S4 Movies). Of the pancake-shaped cells, some migrated persistently albeit more slowly than the acorn-shaped cells, some migrated intermittently, and some did not migrate at all. The pancake-shaped morphology was most frequent on periostin or TGFBI coated at 10 μg/ml (Fig 3C). Pancake-shaped cells eosinophils had a mean area of 199 μm^2^, which was significantly higher than that of acorn-shaped cells (Fig 3D). In addition, a few cells were observed alternating between the acorn- and pancake-shaped morphologies, transitioning back from pancake- to acorn-shaped (also see S1–S4 Movies).

## Discussion

The widely expressed ECM protein TGFBI, which is a paralog of periostin and induced by TGF-β in several cell types [1, 9], is shown to support specific adhesion and migration of blood eosinophils stimulated by IL-5 family cytokines IL-5, IL-3, or GM-CSF. Eosinophil adhesion was sensitive to the coating concentration of TGFBI and concentration of the activating cytokine. Effects of coating concentration was similar for TGFBI and periostin, indicating that the adhesive site(s) reside(s) somewhere in the homologous region encompassing the N-terminal cysteine-rich sequence and the four FAS1 modules shared by the two proteins [1]. Eosinophil adhesion to TGFBI was mediated by α_M_β_2_ integrin, for which the highly activated conformation, reported by mAb CBRM1/5, is known to be induced by IL-5 or IL-3 [17–21]. The dependence on α_M_β_2_ for adhesion of eosinophils to TGFBI is consistent with the report that TGFBI supports monocyte adhesion in an α_M_β_2_-dependent manner [13]. The present adhesion results indicate that the paralogs TGFBI and periostin support adhesion of eosinophils by similar mechanisms. Immobilized TGFBI also significantly supported random motility of cytokine-stimulated eosinophils. However, TGFBI was less effective than PN-L or PN-S, and PN-S was less effective than PN-L in supporting migration. These quantitative differences in motility on TGFBI, PN-S, and PN-L implicate the alternatively spliced sequences in the C-terminal tail of periostin [1] as being important for maximal migration. Thus, in addition to (an) adhesive site(s) in the major and conserved part of TGFBI and periostin, there appears to be another, pro-migratory, site in full-length periostin’s tail.

GM-CSF was the most potent cytokine for adhesion to TGFBI at 1 h, being active at a lower concentration than IL-5 or IL-3. The potency may be related to the greater abundance of CS2RA (GM-CSF receptor α) in comparison to IL3RA (IL-3 receptor α) and IL5RA (IL-5 receptor α) subunits in blood eosinophils at baseline, as found by quantitative proteomic analysis [22]. IL-3 supported greater eosinophil motility over 20 h on TGFBI or periostin (PN-L) than IL-5 or GM-CSF. This is in line with the observation that IL-3 upregulates the cell-surface expression of and induces the high-activity conformation of α_M_β_2_ integrin at 20 h to a significantly greater degree than does IL-5 [21]. The differential effect between the cytokines may in turn be a function of the fact that IL-3 upregulates the cell-surface level of its cognate IL-3 receptor α subunit (IL3RA), whereas IL-5 downregulates IL-5 receptor α (IL5RA) at 4–24 h [20, 23–27]. Overall, the greater effect by IL-3 at 20 h is consistent with the scenario that IL-3 induces stronger and prolonged signaling and translation in eosinophils than does IL-5 or GM-CSF [21, 25, 28–30]. Our results hint at important differences among IL-5 family cytokines in their effects on eosinophil adhesion and migration at various times after eosinophil activation. IL-5, IL-3, and GM-CSF are present in bronchoalveolar lavage (BAL) fluid from subjects with mild allergic asthma and are increased after segmental lung antigen challenge [31]. IL-5 is produced by several cell types including T helper cell type 2 (TH_2_) cells, mast cells, NK cells, and type 2 innate lymphoid cells; IL-3 is produced by, e.g., T cells, macrophages, stromal cells, NK cells, and mast cells; and GM-CSF by a variety of cells such as T cells, macrophages, endothelial cells, fibroblasts, and epithelial cells [32, 33]. The greater effect by GM-CSF and IL-3 than IL-5 on eosinophil adhesion at 1 h and migration over 20 h, respectively, may motivate therapeutic targeting of GM-CSF or IL-3, or their receptors, in addition to or as an alternative to IL-5 or its receptor, in order to inhibit activation of eosinophils and their interactions with matrix in tissues in eosinophilic diseases.

Video microscopy revealed that periostin and TGFBI support two modes of migration by IL-5 family cytokine-activated eosinophils. The majority of migrating eosinophils had, at least during the first 35 minutes after activation with IL-5, a polarized acorn-shaped morphology with a ruffled forward edge and migrated rapidly and persistently with granules closely clustered together, in front of the nucleus. This morphology tended to be most frequent and thus appears to be favored on periostin (PN-L) coated at 5 μg/ml. Cells with the acorn-shaped morphology had a mean area of 108 μm^2^. In addition, this morphology is similar to that of newt (*Taricha granulosa*) eosinophils migrating on quartz coverslips in the presence of newt serum; the newt eosinophils were observed to be tear-drop shaped and polarized, crawling with an advancing lamellipod and the nucleus located near the other, rear end [34, 35]. Some eosinophils in our experiments adopted a flattened pancake-shaped morphology with dispersed granules and nuclear lobes, and slower migration. This morphology was most frequent on a protein coating concentration of 10 μg/ml. Eosinophils with the pancake-shaped morphology had a mean area of 199 μm^2^, significantly greater than and almost twice as large as the acorn-shaped cells. A few cells converted between the two morphologies transitioning back from pancake- to acorn-shaped. We do not know how much time the eosinophils spend in either mode or morphology during a longer time, e.g., during 20 h, which was the incubation time in the bead-clearing motility assay. The acorn-shaped morphology appears to be somewhat similar to that described for migrating neutrophils [36], except that in the acorn-shaped migrating eosinophils, the nucleus is localized at the rear end of the cell, a localization similar to that in suspended acutely IL-5 activated eosinophils [16], whereas in migrating or suspended activated neutrophils the nucleus has a random localization [16, 36].

In the lung, TGFBI appears to be produced by resident cells and is localized to the vascular and airway ECM [10]. TGF-β is synthesized by a large variety of cells, including epithelial cells, fibroblasts, and immune cells [32], is produced in various disease states, and is believed to contribute to tissue remodeling, e.g., in the airway in asthma [37–40]. Eosinophils themselves, which contain TGF-β1 [22], are believed to be one source of TGF-β in eosinophilic disorders [37]. Periostin is upregulated by IL-13 in bronchial epithelial cells and lung fibroblasts and deposited widely in the bronchi of subjects with asthma [1, 41–44]. IL-13, which is secreted by, e.g., TH_2_ cells [2], acts on epithelial cells to cause basal secretion of periostin [42]. Periostin is not detected or detected at very low levels (pg/ml) in bronchoalveolar lavage from normal or asthmatic subjects, indicating that secretion of periostin into the bronchial lumen does not occur or is negligible [45]. In addition, periostin can be secreted by stromal cells stimulated by TGF-β and other cytokines and growth factors at sites of injury or inflammation [41, 46–48]. It should be emphasized, however, that little is known about how TGFBI or periostin is deposited in the ECM [1], and therefore caution should be taken in extrapolating studies done on interactions of eosinophils with coatings of these proteins to events that may take place in the lung. It should also be noted that nine splice variants of periostin have been identified, only two of which were tested by us. Eight splice variants have been detected in normal fetal lung, including the longest isoform and isoforms lacking 1–4 of exons 17, 18, 19, and 21, whereas five variants, not including the longest one, were found in adult lung [49, 50]. In idiopathic pulmonary fibrosis (IPF), exon 21 was discovered to be more likely spliced out than in controls [51]. Nevertheless, the findings lead us to speculate that eosinophils move rapidly by haptotaxis up a gradient of adhesive ECM ligand in the form of periostin or possibly TGFBI and, when having reached a site with higher density of ligand, slow down to surveil and “decide” whether to reside quietly in the tissue or release effectors.

## Supporting information

S1 Movie DIC video microscopy of eosinophils migrating on periostin coated at 5 μg/ml. PN-L (longest periostin variant), 20–35 minutes after addition of IL-5, 50 ng/ml. Representative experiment of five.
(MOV)

S2 Movie DIC video microscopy of eosinophils migrating on periostin coated at 10 μg/ml. PN-L, 20–35 minutes after addition of IL-5, 50 ng/ml. Representative experiment of five.
(MOV)

S3 Movie DIC video microscopy of eosinophils migrating on TGFBI coated at 5 μg/ml. R&D TGFBI, 20–35 minutes after addition of IL-5, 50 ng/ml. Representative experiment of five.
(MOV)

S4 Movie DIC video microscopy of eosinophils migrating on TGFBI coated at 10 μg/ml. R&D TGFBI, 20–35 minutes after addition of IL-5, 50 ng/ml. Representative experiment of five.
(MOV)

## References

[1] Mosher DF, Johansson MW, Gillis ME, Annis DS. Periostin and TGF-beta-induced protein: Two peas in a pod? Crit Rev Biochem Mol Biol. 2015;50(5):427–39. 10.3109/10409238.2015.1069791 ; PubMed Central PMCID: PMC4606919. 26288337 PMC4606919

[2] Doran E, Cai F, Holweg CT, Wong K, Brumm J, Arron JR. Interleukin-13 in asthma and other eosinophilic disorders. Front Med. 2017;4:139 10.3389/fmed.2017.00139 Pubmed Central PMCID: PMC5627038. 29034234 PMC5627038

[3] Johansson MW, Annis DS, Mosher DF. Alpha(M)beta(2) integrin-mediated adhesion and motility of IL-5-stimulated eosinophils on periostin. Am J Respir Cell Mol Biol. 2013;48(4):503–10. 10.1165/rcmb.2012-0150OC ; PubMed Central PMCID: PMC3653603. 23306834 PMC3653603

[4] Johansson MW, Khanna M, Bortnov V, Annis DS, Nguyen CL, Mosher DF. IL-5-stimulated eosinophils adherent to periostin undergo stereotypic morphological changes and ADAM8-dependent migration. Clin Exp Allergy. 2017;47(10):1263–74. 10.1111/cea.12934 ; PubMed Central PMCID: PMC5623171. 28378503 PMC5623171

[5] Wang M, Wang X, Zhang N, Wang H, Li Y, Fan E, et al Association of periostin expression with eosinophilic inflammation in nasal polyps. J Allergy Clin Immunol. 2015;136(6):1700–3 e9. 10.1016/j.jaci.2015.09.005 . 26521039

[6] Noguchi T, Nakagome K, Kobayashi T, Uchida Y, Soma T, Nakamoto H, et al Periostin upregulates the effector functions of eosinophils. J Allergy Clin Immunol. 2016;138(5):1449–52 e5. 10.1016/j.jaci.2016.05.020 . 27423493

[7] Johansson MW, Evans MD, Crisafi GM, Holweg CT, Matthews JG, Jarjour NN. Serum periostin is associated with type 2 immunity in severe asthma. J Allergy Clin Immunol. 2016;137(6):1904–7 e2. 10.1016/j.jaci.2015.12.1346 ; PubMed Central PMCID: PMC5580680. 27061252 PMC5580680

[8] Lukassen MV, Scavenius C, Thogersen IB, Enghild JJ. Disulfide bond pattern of transforming growth factor beta-induced protein. Biochemistry. 2016;55(39):5610–21. 10.1021/acs.biochem.6b00694 . 27609313

[9] Thapa N, Lee BH, Kim IS. TGFBIp/betaig-h3 protein: a versatile matrix molecule induced by TGF-beta. Int J Biochem Cell Biol. 2007;39(12):2183–94. 10.1016/j.biocel.2007.06.004 . 17659994

[10] Billings PC, Herrick DJ, Howard PS, Kucich U, Engelsberg BN, Rosenbloom J. Expression of betaig-h3 by human bronchial smooth muscle cells: localization To the extracellular matrix and nucleus. Am J Respir Cell Mol Biol. 2000;22(3):352–9. 10.1165/ajrcmb.22.3.3732 . 10696072

[11] Ahlfeld SK, Wang J, Gao Y, Snider P, Conway SJ. Initial suppression of transforming growth factor-beta signaling and loss of TGFBI causes early alveolar structural defects resulting in bronchopulmonary dysplasia. Am J Pathol. 2016;186(4):777–93. 10.1016/j.ajpath.2015.11.024 ; PubMed Central PMCID: PMC5808152. 26878215 PMC5808152

[12] Schwanekamp JA, Lorts A, Sargent MA, York AJ, Grimes KM, Fischesser DM, et al TGFBI functions similar to periostin but is uniquely dispensable during cardiac injury. PLoS One. 2017;12(7):e0181945 10.1371/journal.pone.0181945 ; PubMed Central PMCID: PMC5531541. 28750100 PMC5531541

[13] Kim HJ, Kim IS. Transforming growth factor-beta-induced gene product, as a novel ligand of integrin alphaMbeta2, promotes monocytes adhesion, migration and chemotaxis. Int J Biochem Cell Biol. 2008;40(5):991–1004. Epub 2007/12/18. 10.1016/j.biocel.2007.11.001 . 18083624

[14] Mosher DF, Huwiler KG, Misenheimer TM, Annis DS. Expression of recombinant matrix components using baculoviruses. Methods Cell Biol. 2002;69(3):69–81. . 12071009 10.1016/s0091-679x(02)69008-9

[15] Annis DS, Ma H, Balas DM, Kumfer KT, Sandbo N, Potts GK, et al Absence of vitamin K-dependent gamma-carboxylation in human periostin extracted from fibrotic lung or secreted from a cell line engineered to optimize gamma-carboxylation. PLoS One. 2015;10(8):e0135374 10.1371/journal.pone.0135374 ; PubMed Central PMCID: 4537219. 26273833 PMC4537219

[16] Han ST, Mosher DF. IL-5 induces suspended eosinophils to undergo unique global reorganization associated with priming. Am J Respir Cell Mol Biol. 2014;50(3):654–64. 10.1165/rcmb.2013-0181OC ; PubMed Central PMCID: PMC4068932. 24156300 PMC4068932

[17] Barthel SR, Jarjour NN, Mosher DF, Johansson MW. Dissection of the hyperadhesive phenotype of airway eosinophils in asthma. Am J Respir Cell Mol Biol. 2006;35(3):378–86. 10.1165/rcmb.2006-0027OC ; PubMed Central PMCID: PMC1550734. 16601240 PMC1550734

[18] Barthel SR, Johansson MW, McNamee DM, Mosher DF. Roles of integrin activation in eosinophil function and the eosinophilic inflammation of asthma. J Leukoc Biol. 2008;83(1):1–12. 10.1189/jlb.0607344 ; PubMed Central PMCID: PMC2859217. 17906117 PMC2859217

[19] Johansson MW, Mosher DF. Integrin activation states and eosinophil recruitment in asthma. Front Pharmacol. 2013;4:33 10.3389/fphar.2013.00033 ; PubMed Central PMCID: PMC3612688. 23554594 PMC3612688

[20] Johansson MW. Activation states of blood eosinophils in asthma. Clin Exp Allergy. 2014;44(4):482–98. 10.1111/cea.12292 ; PubMed Central PMCID: PMC4057046. 24552191 PMC4057046

[21] Esnault S, Johansson MW, Kelly EA, Koenderman L, Mosher DF, Jarjour NN. IL-3 up-regulates and activates human eosinophil CD32 and alphaMbeta2 integrin causing degranulation. Clin Exp Allergy. 2017;47(4):488–98. 10.1111/cea.12876 ; PubMed Central PMCID: PMC5378663. 28000949 PMC5378663

[22] Wilkerson EM, Johansson MW, Hebert AS, Westphall MS, Mathur SK, Jarjour NN, et al The peripheral blood eosinophil proteome. J Proteome Res. 2016;15(5):1524–33. 10.1021/acs.jproteome.6b00006 ; PubMed Central: PMCID: PMC5222579. 27005946 PMC5222579

[23] Liu LY, Sedgwick JB, Bates ME, Vrtis RF, Gern JE, Kita H, et al Decreased expression of membrane IL-5 receptor alpha on human eosinophils: II. IL-5 down-modulates its receptor via a proteinase-mediated process. J Immunol. 2002;169(11):6459–66. . 12444155 10.4049/jimmunol.169.11.6459

[24] Gregory B, Kirchem A, Phipps S, Gevaert P, Pridgeon C, Rankin SM, et al Differential regulation of human eosinophil IL-3, IL-5, and GM-CSF receptor alpha-chain expression by cytokines: IL-3, IL-5, and GM-CSF down-regulate IL-5 receptor alpha expression with loss of IL-5 responsiveness, but up-regulate IL-3 receptor alpha expression. J Immunol. 2003;170(11):5359–66. . 12759409 10.4049/jimmunol.170.11.5359

[25] Esnault S, Kelly EA, Johansson MW, Liu LY, Han ST, Akhtar M, et al Semaphorin 7A is expressed on airway eosinophils and upregulated by IL-5 family cytokines. Clin Immunol. 2014;150(1):90–100. 10.1016/j.clim.2013.11.009 ; PubMed Central PMCID: PMC3947215. 24333536 PMC3947215

[26] Yoshimura-Uchiyama C, Yamaguchi M, Nagase H, Matsushima K, Igarashi T, Iwata T, et al Changing expression of IL-3 and IL-5 receptors in cultured human eosinophils. Biochem Biophys Res Comm. 2003;309(1):26–31. . 12943658 10.1016/s0006-291x(03)01526-2

[27] Esnault S, Hebert AS, Jarjour NN, Coon JJ, Mosher DF. Proteomic and phosphoproteomic changes induced by prolonged activation of human eosinophils with IL-3. J Proteome Res. 2018;17(6):2102–11. Epub 2018/05/01. 10.1021/acs.jproteome.8b00057 ; PubMed Central PMCID: PMC5984179. 29706072 PMC5984179

[28] Esnault S, Kelly EA, Shen ZJ, Johansson MW, Malter JS, Jarjour NN. IL-3 maintains activation of the p90S6K/RPS6 pathway and increases translation in human eosinophils. J Immunol. 2015;195(6):2529–39. 10.4049/jimmunol.1500871 ; PubMed Central PMCID: PMC4561194. 26276876 PMC4561194

[29] Esnault S, Kelly EA. Essential mechanisms of differential activation of eosinophils by IL-3 compared to GM-CSF and IL-5. Crit Rev Immunol. 2016;36(5):429–44. 10.1615/CritRevImmunol.2017020172 ; PubMed Central PMCID: PMC5586489. 28605348 PMC5586489

[30] Esnault S, Shen ZJ, Malter JS. Protein translation and signaling in human eosinophils. Front Med. 2017;4:150 10.3389/fmed.2017.00150 ; PubMed Central PMCID: PMC5609579. 28971096 PMC5609579

[31] Johansson MW, Kelly EA, Busse WW, Jarjour NN, Mosher DF. Up-regulation and activation of eosinophil integrins in blood and airway after segmental lung antigen challenge. J Immunol. 2008;180(11):7622–35. ; PubMed Central PMCID: PMC2585992. 18490765 10.4049/jimmunol.180.11.7622 PMC2585992

[32] Akdis M, Aab A, Altunbulakli C, Azkur K, Costa RA, Crameri R, et al Interleukins (from IL-1 to IL-38), interferons, transforming growth factor beta, and TNF-alpha: Receptors, functions, and roles in diseases. J Allergy Clin Immunol. 2016;138(4):984–1010. 10.1016/j.jaci.2016.06.033 . 27577879

[33] Shiomi A, Usui T. Pivotal roles of GM-CSF in autoimmunity and inflammation. Mediators Inflamm. 2015;2015:568543 10.1155/2015/568543 ; PubMed Central PMCID: PMC4370199. 25838639 PMC4370199

[34] Koonce MP, Cloney RA, Berns MW. Laser irradiation of centrosomes in newt eosinophils: evidence of centriole role in motility. J Cell Biol. 1984;98:1999–2010. ; PubMed Central PMCID: PMC2113065. 6725407 10.1083/jcb.98.6.1999 PMC2113065

[35] Gilbert SH, Perry K, Fay FS. Mediation of chemoattractant-induced changes in [Ca^2+^]_i_ and cell shape, polarity, and locomotion by InsP_3_, DAG, and protein kinase C in newt eosinophils. J Cell Biol. 1994;127:489–503. ; PubMed Central PMCID: PMC2120201. 7929591 10.1083/jcb.127.2.489 PMC2120201

[36] Hind LE, Vincent WJ, Huttenlocher A. Leading from the back: The role of the uropod in neutrophil polarization and migration. Dev Cell. 2016;38(2):161–9. 10.1016/j.devcel.2016.06.031 ; PubMed Central PMCID: PMC4982870. 27459068 PMC4982870

[37] McBrien CN, Menzies-Gow A. The biology of eosinophils and their role in asthma. Front Med. 2017;4:93 10.3389/fmed.2017.00093 ; PubMed Central PMCID: PMC5491677. 28713812 PMC5491677

[38] Venge P. The eosinophil and airway remodelling in asthma. Clin Respir J. 2010;4 Suppl 1:15–9. 10.1111/j.1752-699X.2010.00192.x . 20500605

[39] Nhu QM, Aceves SS. Tissue remodeling in chronic eosinophilic esophageal inflammation: Parallels in asthma and therapeutic perspectives. Front Med. 2017;4:128 10.3389/fmed.2017.00128 ; PubMed Central PMCID: PMC5549614. 28831387 PMC5549614

[40] Halwani R, Al-Muhsen S, Al-Jahdali H, Hamid Q. Role of transforming growth factor-beta in airway remodeling in asthma. Am J Respir Cell Mol Biol 2011;44(2):127–33. 10.1165/rcmb.2010-0027TR . 20525803

[41] Conway SJ, Izuhara K, Kudo Y, Litvin J, Markwald R, Ouyang G, et al The role of periostin in tissue remodeling across health and disease. Cell Mol Life Sci. 2014;71(7):1279–88. 10.1007/s00018-013-1494-y ; PubMed Central PMCID: PMC3949008. 24146092 PMC3949008

[42] Sidhu SS, Yuan S, Innes AL, Kerr S, Woodruff PG, Hou L, et al Roles of epithelial cell-derived periostin in TGF-beta activation, collagen production, and collagen gel elasticity in asthma. Proc Natl Acad Sci USA. 2010;107(32):14170–5. Epub 2010/07/28. 10.1073/pnas.1009426107 ; PubMed Central PMCID: PMC2922596. 20660732 PMC2922596

[43] Takayama G, Arima K, Kanaji T, Toda S, Tanaka H, Shoji S, et al Periostin: a novel component of subepithelial fibrosis of bronchial asthma downstream of IL-4 and IL-13 signals. J Allergy Clin Immunol. 2006;118(1):98–104. Epub 2006/07/04. 10.1016/j.jaci.2006.02.046 . 16815144

[44] Woodruff PG, Boushey HA, Dolganov GM, Barker CS, Yang YH, Donnelly S, et al Genome-wide profiling identifies epithelial cell genes associated with asthma and with treatment response to corticosteroids. Proc Natl Acad Sci USA. 2007;104(40):15858–63. Epub 2007/09/28. 10.1073/pnas.0707413104 ; PubMed Central PMCID: PMC2000427. 17898169 PMC2000427

[45] Nakamura Y, Nagashima H, Ohta S, Ono J, Yamauchi K, Izuhara K. Periostin in the bronchial lavage fluid of asthma patients. Allergol Int. 2015;64(2):209–10. Epub 2015/04/04. 10.1016/j.alit.2015.01.001 . 25838104

[46] Liu AY, Zheng H, Ouyang G. Periostin, a multifunctional matricellular protein in inflammatory and tumor microenvironments. Matrix Biol. 2014;37:150–6. 10.1016/j.matbio.2014.04.007 . 24813586

[47] Izuhara K, Nunomura S, Nanri Y, Ogawa M, Ono J, Mitamura Y, et al Periostin in inflammation and allergy. Cell Mol Life Sci. 2017;74(23):4293–303. 10.1007/s00018-017-2648-0 . 28887633 PMC11107676

[48] Izuhara K, Conway SJ, Moore BB, Matsumoto H, Holweg CT, Matthews JG, et al Roles of periostin in respiratory disorders. Am J Respir Crit Care Med. 2016;193(9):949–56. 10.1164/rccm.201510-2032PP ; PubMed Central PMCID: PMC4872656. 26756066 PMC4872656

[49] Morra L, Rechsteiner M, Casagrande S, von Teichman A, Schraml P, Moch H, et al Characterization of periostin isoform pattern in non-small cell lung cancer. Lung Cancer. 2012;76(2):183–90. 10.1016/j.lungcan.2011.10.013 . 22079858

[50] Viloria K, Hill NJ. Embracing the complexity of matricellular proteins: the functional and clinical significance of splice variation. Biomol Concepts. 2016;7(2):117–32. 10.1515/bmc-2016-0004 . 27135623

[51] Nance T, Smith KS, Anaya V, Richardson R, Ho L, Pala M, et al Transcriptome analysis reveals differential splicing events in IPF lung tissue. PLoS One. 2014;9(3):e92111 10.1371/journal.pone.0092111 ; PubMed Central PMCID: PMC3960165. 24647608 PMC3960165

